# Heterologous Expression of the Nitrogen-Fixing Gene Cluster from *Paenibacillus polymyxa* in *Bacillus subtilis*

**DOI:** 10.3390/microorganisms13061320

**Published:** 2025-06-06

**Authors:** Xiuling Wang, Shiqing Gao, Jun Fu, Ruijuan Li

**Affiliations:** State Key Laboratory of Microbial Technology, Shandong University, Qingdao 266237, China; 18706380569@163.com (X.W.); gaoshiqing8618@163.com (S.G.)

**Keywords:** nitrogen-fixing gene cluster, *Bacillus subtilis*, heterologous expression, promoter

## Abstract

Microbially mediated biological nitrogen fixation is pivotal to sustainable agricultural development. However, optimizing nitrogenase activity in native biological nitrogen-fixing bacteria has been hindered by the complexities of genetic manipulation. Heterologous expression has served as a foundational strategy for engineering next-generation nitrogen-fixing microbial agents. In this study, genomic analysis of *Paenibacillus polymyxa* CR1 revealed an 11 kb nitrogen-fixing (*nif*) gene cluster. The *nif* cluster was first synthesized and then assembled using ExoCET technology and finally integrated into the genome of *Bacillus subtilis* 168 via double-exchange recombination. RT-PCR confirmed the transcription of the *nif* cluster; however, no nitrogenase activity was detected in the acetylene reduction assay. A promoter replacement strategy (replacing the native promoter with P_veg_) enabled *B. subtilis* to produce active nitrogenase. However, stronger promoters—namely, P_43_ and P_tp2_—did not further enhance nitrogenase activity. This demonstrates that promoter selection requires balancing transcriptional strength with systemic compatibility, particularly for metalloenzymes demanding precise cofactor assembly. This is the first report describing the heterologous expression of the *nif* gene cluster in *B. subtilis*, establishing a foundation for engineering high-efficiency nitrogen-fixing biofertilizers.

## 1. Introduction

Microbially mediated enzymatic processes drive biological nitrogen fixation, which involves the transformation of atmospheric nitrogen (N_2_) into bioavailable ammonia, a keystone ecological mechanism for sustainable agriculture [1]. This mechanism not only replenishes soil nitrogen reservoirs but also reduces dependence on synthetic fertilizers, thereby alleviating environmental burdens linked to industrial fertilizer production, including greenhouse gas emissions and eutrophication risks [2]. Nitrogen-fixing microorganisms can generally be categorized into three types based on their relationship with plant hosts: symbiotic, free living, and associative [3,4]. Symbiotic nitrogen fixers, exemplified by rhizobia, require root nodule formation with specific leguminous plants, limiting their application in non-leguminous crops [5]. In contrast, free-living and associative nitrogen-fixing bacteria are compatible with a wider range of plant species. Despite their ecological potential, native biological nitrogen-fixing bacteria have historically encountered intractable barriers in agricultural applications due to the complexities of genetic manipulation, which hinders nitrogenase activity optimization, and their limited environmental robustness in field conditions.

Synthetic biology has emerged as a transformative strategy to address these challenges, focusing on transferring native nitrogen-fixing (*nif*) gene clusters into genetically amenable heterologous hosts [6,7,8]. Chen’s group successfully transferred the *nif* cluster of *Paenibacillus polymyxa* WLY78 into *E. coli*. Through gene deletion, they identified a minimal set of nine *nif* genes that were sufficient for synthesizing catalytically active nitrogenase in *E. coli* [9]. However, the resulting enzyme activity remained insufficient to support growth when using dinitrogen as the sole nitrogen source. Notably, Yang and colleagues achieved a breakthrough by leveraging synthetic biology to redesign the *nif* cluster of *Klebsiella oxytoca*. Their approach involved cluster minimization and the balanced expression of essential protein components for nitrogenase biosynthesis and activity [10]. This engineering milestone enabled *E. coli* to sustain growth under nitrogen-limited conditions, marking a critical advancement toward practical agricultural applications.

Although *E. coli* exhibits unique advantages in fundamental research for dissecting nitrogen-fixation mechanisms due to its rapid growth rate and mature genetic manipulation techniques, its restricted ability to colonize plant roots and its weak ecological competitiveness are significant obstacles to its agricultural application. In contrast, *Bacillus subtilis*, as a plant-growth-promoting rhizobacterium (PGPR) [11], demonstrates remarkable agronomic benefits. It effectively promotes plant growth, enhances disease resistance, and exhibits robust soil adaptability [12,13,14]. Additionally, its well-established molecular toolkit [15], including promoters and transformation systems, facilitates customized refactoring of the *nif* gene cluster. All these characteristics strongly suggest that *B. subtilis* serves as a more promising chassis for agricultural use [16]. By genetically engineering nitrogen-fixing *B. subtilis* strains, we aim to develop novel biofertilizers that can synergistically perform nitrogen fixation and provide phytoprotection, thereby offering viable solutions for sustainable agriculture.

This article reports the first successful functional heterologous expression of the *nif* gene cluster from *P. polymyxa* in *B. subtilis*. Genome mining identified an 11 kb *nif* gene cluster (spanning *nifB* to *nifV*) containing nine genes in *P. polymyxa* CR1. Using ExoCET (exonuclease combined with RecET recombination) technology, the cluster was modularly assembled and then subcloned into an integration vector. A double-exchange chromosomal integration strategy was employed to generate the engineered strain *B. subtilis* 168::CR1nif. However, the native promoter-driven cluster exhibited undetectable nitrogenase activity in acetylene reduction assays. Subsequent optimization revealed that replacing the native promoter with the host-derived constitutive promoter P_veg_ restored nitrogenase activity, underscoring the critical role of promoter compatibility in heterologous systems. This breakthrough lays a foundation for the rational design of high-efficiency nitrogen-fixing strains, offering transformative potential for sustainable agriculture and biotechnology.

## 2. Materials and Methods

### 2.1. Strains, Plasmids, and Growth Conditions

The strains and plasmids used in this study are listed in Table S1. *E. coli* was cultured in LB medium (tryptone, 10 g/L; yeast extract, 5 g/L; NaCl, 1 g/L; agar for solid medium, 15 g/L). *B. subtilis* was cultured in LBGS medium (tryptone, 10 g/L; yeast extract, 5 g/L; NaCl, 1 g/L; glucose, 2 g/L; sucrose, 10 g/L; Na_2_HPO_4_·12H_2_O, 2.5 g/L; agar for solid medium, 15 g/L). A nitrogen-limiting medium was used in the nitrogenase activity assay (KH_2_PO_4_, 3.4 g/L; Na_2_HPO_4_, 26.3 g/L; biotin, 10 μg/L; MgSO_4_, 30 mg/L; p-aminobenzoic acid, 10 μg/L; CaCl_2_·2H_2_O, 26 mg/L; ferric citrate, 36 mg/L; MnSO_4_·H_2_O, 0.33 mg/L; Na_2_MoO_4_·2H_2_O, 7.6 mg/L; glucose, 4 g/L). Antibiotics were added when necessary: 15 μg/mL kanamycin, 100 μg/mL ampicillin, and 80 μg/mL spectinomycin.

### 2.2. Assembly of the nif Gene Cluster from P. polymyxa CR1

Four plasmids containing *nif* gene cluster fragments (pUC19-kan-F1 to F4) were synthesized by TsingKe Biotechnology Co., Ltd., Beijing, China. Following large-scale plasmid purification, all plasmids were linearized via BamHI digestion. Concurrently, PCR amplification was performed using the plasmid pBR322-amp-tetR-tetO-ccdB-hyg as a template and the primers pBR322-1/pBR322-2. The four linearized fragments (F1 to F4) and the PCR products (pBR322-amp) were purified using agarose gel electrophoresis. The recovered fragments (per fragment 300 ng) were treated with T4 DNA polymerase (0.13 μL) mixed with Buffer 2.1 (2 μL), with ddH_2_O added to obtain a final volume of 20 μL. The mixture in a PCR tube was placed in a PCR machine for the T4 DNA polymerase reaction. The reaction program was 25 °C for 1 h; 75 °C for 20 min; 50 °C for 30 min; and 4 °C for 10 min. The reaction product was then transferred into competent *E. coli* GB05-dir cells for recombination [17]. Transformants were selected on LB agar plates supplemented with 100 μg/mL ampicillin. Positive recombinants carrying pBR322-amp-CR1nif were validated through restriction analysis using EcoRI, with expected fragment sizes confirming successful assembly.

### 2.3. Integration of the nif Gene Cluster into the Genome of B. subtilis 168

The p15A-ha-spec fragment was amplified from p15A-amyEF-amp-ccdB-spec-amyER using the primers p15A-1 and p15A-2. Following gel purification, the PCR product was co-electroporated with the pBR322-amp-CR1nif plasmid into competent *E. coli* GB05-red cells. Recombinants carrying p15A-ha-spec-CR1nif were selected on LB agar supplemented with 80 μg/mL spectinomycin. Subsequently, the validated p15A-ha-spec-CR1nif plasmid was introduced into *B. subtilis* 168 via electroporation, as previously described [18]. Transformants (named 168-CR1nif) were screened on spectinomycin-containing LBGS plates (80 μg/mL) and confirmed through colony PCR with two primer pairs, F1/F2 and R1/R2, verifying *nif* cluster insertion. The sequences of the primers are shown in Table S2.

### 2.4. Substitution of the nifB Promoter

First, the ampicillin resistance fragment (amp) was amplified from the pR6K-amp-ccdB template using the primers amp-1/amp-2. Simultaneously, the promoter fragment Pveg was amplified via PCR from the genomic DNA of *B. subtilis* 168 with the primers veg-1/veg-2. Through overlap extension PCR, the amp fragment was directionally fused to Pveg, yielding the fragment amp-Pveg. Then, the purified fusion fragment amp-Pveg was co-electroporated with p15A-ha-spec-CR1nif into *E. coli* GB05-red. Recombinants were selected on LB agar containing 100 μg/mL ampicillin and 80 μg/mL spectinomycin, generating the promoter-modified vector p15A-ha-spec-amp-Pveg-CR1nif. Finally, the vector was introduced into *B. subtilis* 168 via electroporation as previously described [18]. Transformant *B. subtilis* 168-P_veg_-CR1nif was selected on LBGS medium supplemented with 80 μg/mL spectinomycin.

Using the primers 43-1 and 43-2, the target fragment (P43) was amplified via PCR from *B. subtilis* 168 genomic DNA. Subsequently, fusion PCR was performed to ligate the fragment P43 with the ampicillin resistance fragment (amp), generating the fragment amp-P43. For the assembly of the amp-Ptp2 fragment, the initial amp-tp2-1 fragment was first amplified using the amp-1/tp2-2 primers and the pR6K-amp-ccdB template. A second PCR with the primers amp-1/tp2-3 and the amp-tp2-1 fragment as the template yielded the final amp-Ptp2 fragment. After purification, both amp-P43 and amp-Ptp2 were used for vector and strain construction using the same experimental procedures as those used for amp-Pveg. All primer sequences are shown in Table S2.

### 2.5. Reverse Transcription Quantitative PCR (RT-qPCR) Analysis

The Tiangen RNAprep Pure Cell/Bacteria Kit (Tiangen Biotech Co., Ltd., Beijing, China, Cat. No. DP430) was used to extract total RNA from bacterial cultures that had been cultivated in a nitrogen-limiting medium supplemented with 2 mM glutamate for 16 h. The extracted RNA was subsequently reverse-transcribed into cDNA using the PrimeScript™ RT Reagent Kit containing gDNA Eraser (Perfect Real Time, Takara Bio, Kusatsu, Japan, Code No. RR047A). Quantitative real-time PCR (qPCR) was performed with TB Green^®^ Premix Ex Taq™ II (Tli RNaseH Plus, Takara Bio, Code No. RR82LR) using cDNA as a template. The gene-specific primers (nifB-1/nifB-2, nifK-1/nifK-2, nifV-1/nifV-2, and gyrA-1/gyrA-2) listed in Table S2 were employed to quantify the transcript levels of *nifB*, *nifK*, and *nifV*, with *gyrA* serving as the endogenous reference gene.

### 2.6. Acetylene Reduction Assays

Single colonies were picked from LBGS agar plates and inoculated into 50 mL of fresh LBGS liquid medium, followed by incubation at 30 °C with 250 rpm shaking for 12 h. Bacterial cells were harvested, washed three times with 20 mL of ddH_2_O, and resuspended in a nitrogen-limiting medium supplemented with 2 mM glutamate to adjust the strain density OD_600_ to 0.2. A 20 mL aliquot of the cell suspension was transferred into a 100 mL sealed flask. Anaerobic conditions were established by purging the flask with argon gas (containing 1% O_2_) for 5 min. The culture was incubated at 30 °C with 200 rpm shaking for 12 h, followed by the injection of 10 mL of high-purity acetylene gas. The system was further incubated statically for 72 h. For the acetylene reduction assay (ARA), three independent biological replicates were performed for each experimental condition, with three technical replicates per biological replicate. The headspace gas (1 mL) was sampled using a gas-tight syringe and analyzed using gas chromatography to quantify ethylene production. Nitrogenase activity is quantified as C_2_H_4_ production per hour (nmol/h) by bacterial culture. Error bars represent standard deviations (SDs) of biological replicates. Statistical significance was determined using one-way ANOVA with Tukey’s post hoc test (*p* < 0.05).

## 3. Results and Discussion

### 3.1. The Assembly of the Nitrogen-Fixing Gene Cluster from P. polymyxa CR1

**Figure 1:**
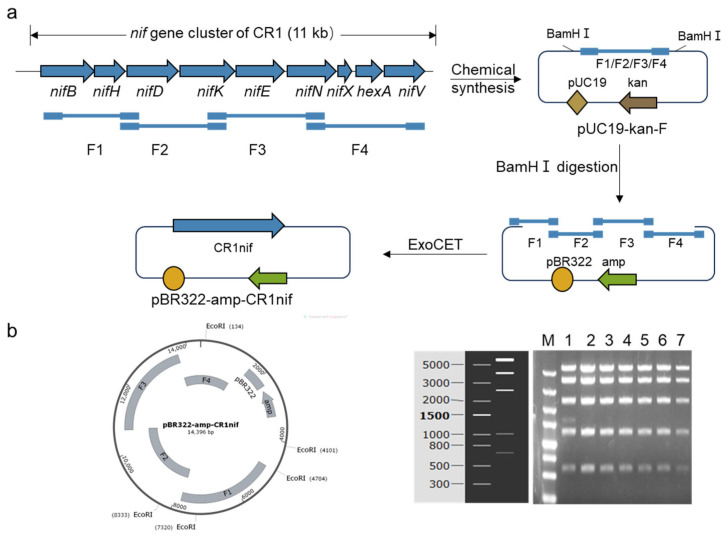
Construction of the cloning vector pBR322-amp-CR1nif harboring the nitrogen-fixing (*nif*) gene cluster of *P. polymyxa* CR1. (**a**) A diagram of the construction process of the vector pBR322-amp-CR1nif. Four fragments (F1, F2, F3, and F4) derived from the *nif* gene cluster were chemically synthesized into the pUC19-kan vector. Following the *Bam*HI digestion of the vector, these fragments were assembled into the pBR322-amp backbone via ExoCET technology, resulting in the final vector: pBR322-amp-CR1nif. (**b**) *Eco*RΙ restriction analysis of the vector pBR322-amp-CR1nif. Lanes 2–7 show complete concordance with the predicted molecular size markers. M: DL 5000 DNA marker.

The genomic analysis of *P. polymyxa* CR1 (GenBank accession No. CP006941) revealed an approximately 11 kb nitrogen-fixing (*nif*) gene cluster, which consists of nine functional genes (*nifB*-*nifV*) (Figure 1a). At the core of this cluster, the *nifHDK* genes are responsible for encoding the MoFe-nitrogenase that plays a key role in nitrogen fixation. Meanwhile, the *nifBENXV* genes are involved in the synthesis and maturation of the MoFe cofactor. Moreover, the gene *hesA* encodes an NAD/FAD-binding protein that participates in the biosynthesis of molybdopterin (a precursor of molybdenum cofactor) and thiamine [19].

In the absence of the original *P. polymyxa* CR1 strain, a synthetic biology strategy was employed for the de novo reconstruction of the *nif* gene cluster. The sequence data of the gene cluster were used to systematically divide the cluster into four overlapping DNA fragments (F1, F2, F3, and F4) (Figure 1a). These fragments were chemically synthesized and individually cloned into the pUC19-kan vector backbone, creating an intermediate plasmid library. Subsequently, the four DNA fragments (F1–F4) were precisely assembled into the pBR322-amp vector through pre-designed homologous arms utilizing ExoCET technology [17], resulting in the generation of a plasmid harboring the complete *nif* gene cluster (Figure 1a). The structural integrity of the plasmid and the sequence accuracy of the gene cluster were confirmed through restriction digestion analysis (Figure 1b) and Sanger sequencing validation.

### 3.2. Integration of the Nitrogen-Fixing Gene Cluster from P. polymyxa CR1 into B. subtilis 168

**Figure 2:**
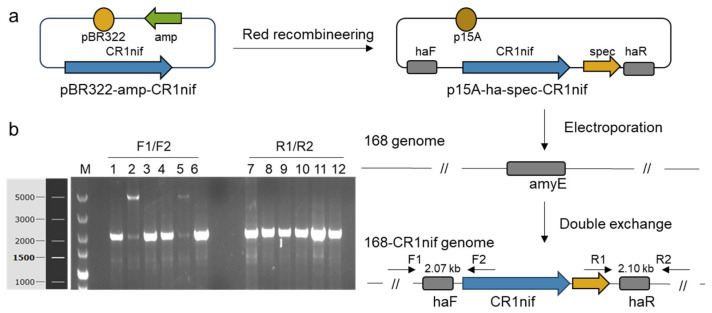
Integration of the *nif* gene cluster into the genome of *B. subtilis* 168. (**a**) A schematic diagram of the construction of *B. subtilis* 168-CR1nif. pBR322-amp-CR1nif: the cloning vector with the pBR322 origin, the ampicillin resistance gene *amp*, and the *nif* cluster; p15A-ha-spec-CR1nif: the integrative vector containing the p15A origin, the amyE-homologous arms haF/haR, the spectinomycin resistance gene *spec*, and the *nif* cluster. The integrative vector was electroporated into *B. subtilis* 168 to achieve genomic integration of the *nif* gene cluster at the *amyE* locus via a double-exchange strategy. F1/F2 and R1/R2 are two pairs of primers used to confirm the integration of the gene cluster. (**b**) Colony PCR verification of genomic integration. Six recombinants were subjected to dual-primer verification. Lanes 1–6: haF-specific amplification with F1/F2 primers; Lanes 7–12: haR-specific amplification with R1/R2 primers. The concurrent detection of both F- and R-terminal amplicons in Lane 1 (F-amplification) and Lane 7 (R-amplification) from the same recombinant confirms successful double-exchange recombination. M: DL5000 DNA marker.

To achieve the precise integration of the *nif* gene cluster into the genome of *B. subtilis* 168 (GenBank accession No. AL0091236), this study adopted a double-exchange strategy. The *nif* cluster was subcloned from the pBR322-amp-CR1nif vector into the integration vector p15A-ha-spec-CR1nif (Figure 2a), which harbored homologous recombination arms (HAs) that targeted the *amyE* locus (GenBank: NC_000964.3). The *amyE* locus was chosen as a neutral integration site because it does not play an essential role in the viability of the host, which was previously verified [20]. The integration vector was introduced into *B. subtilis* 168 via transformation, and colony PCR verification was carried out using two pairs of specific primers: F1/F2 and R1/R2. The results confirmed the occurrence of successful dual homologous recombination events (Figure 2b). The engineered strain harboring the complete *nif* cluster was named *B. subtilis* 168-CR1nif.

### 3.3. Transcription of the nif Gene Cluster in B. subtilis

**Figure 3:**
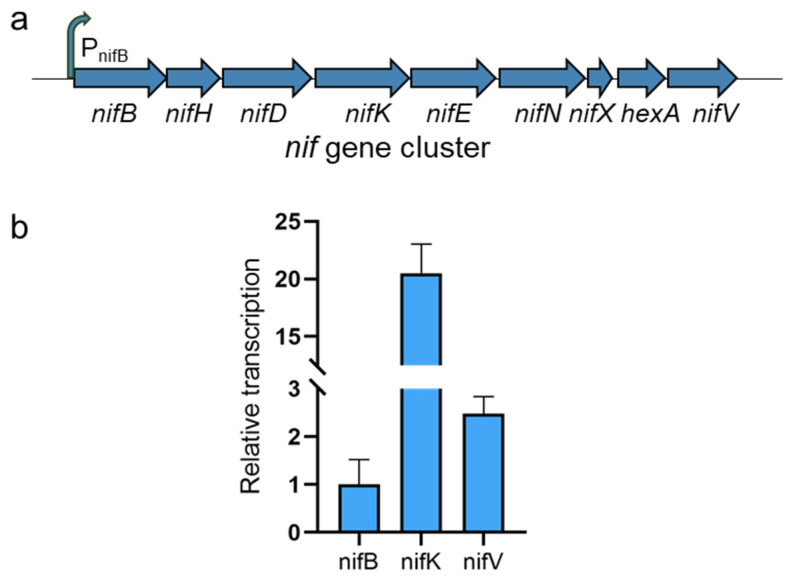
Transcription of the *nif* genes in *B. subtilis* 168. (**a**) The *nif* cluster (*nifB*–*nifV*) under the control of the native promoter P_nifB_. (**b**) The relative transcript levels of *nifB*, *nifK*, and *nifV* in *B. subtilis* 168-CR1nif. The *y*-axis states “Relative transcript level (normalized to *nifB*)” to clarify that the expression levels of *nifK* and *nifV* are expressed as fold changes relative to *nifB*. Data were normalized to *nifB* (set as 1.0) to compare relative expression levels within the *nif* gene cluster. Error bars represent SDs from three biological replicates. The housekeeping gene *gyrA* was used for Ct value standardization across samples.

The transcriptional profile of the *nif* gene cluster was systematically analyzed using real-time quantitative reverse transcription PCR (RT-qPCR), with *gyrA* (DNA gyrase subunit A) serving as the endogenous reference. Despite the nine genes in this cluster being co-transcribed as a polycistronic operon [9], their expression levels exhibited significant deviations from the canonical polar gradient pattern, where promoter-proximal genes typically display higher expression. Notably, as shown in Figure 3b, *nifB* (proximal to the promoter) demonstrated markedly lower transcript abundance compared to the downstream structural gene *nifK*. This inverse correlation challenges the conventional model of operon transcription. The *nifB* gene participates in the biosynthesis and assembly of the MoFe cofactor, while *nifDK* encodes the α_2_β_2_ heterotetrameric MoFe protein, forming the nitrogenase complex with the Fe protein (*nifH*). Collectively, these data suggest that expression divergence may arise from (i) differential stoichiometric demands: MoFe-co biosynthesis genes (e.g., *nifB*) demand lower protein stoichiometry, while structural genes (e.g., *nifDK*) necessitate sustained high expression to maintain enzymatic efficiency; (ii) post-transcriptional regulation: potential mechanisms, such as mRNA stability or translational coupling, may compensate for promoter-proximal transcript suppression.

To address the transcript-level disparity between *nifB* and *nifK*, we performed comprehensive secondary structure predictions for the entire gene cluster and individual coding regions using RNAfold 2.6.3 [21] (Figures S1–S3). The analysis revealed a significantly lower minimum free energy (MFE) for *nifK* (−569.00 kcal/mol) than for *nifB* (−527.10 kcal/mol), indicating higher thermodynamic stability in the *nifK* mRNA secondary structure. This structural stability likely enhances resistance to ribonuclease degradation, leading to increased *nifK* transcript accumulation—consistent with its elevated abundance in RT-PCR assays. The thermodynamic ensemble free energy of *nifK* (−591.73 kcal/mol) further confirmed its improved global structural stability compared to *nifB* (−549.40 kcal/mol). Such energy minimization reduces accessibility to sites for enzymatic cleavage as compact RNA folds sterically hinder RNase binding. Additionally, *nifK*’s higher ensemble diversity (437.88 vs. 380.21) suggests a broader conformational landscape where transiently protected states could shield degradation-prone regions (e.g., ribosome-binding sites or AU-rich motifs), reinforcing transcript resilience.

### 3.4. Heterologous Expression of the nif Gene Cluster in B. subtilis 168

**Figure 4:**
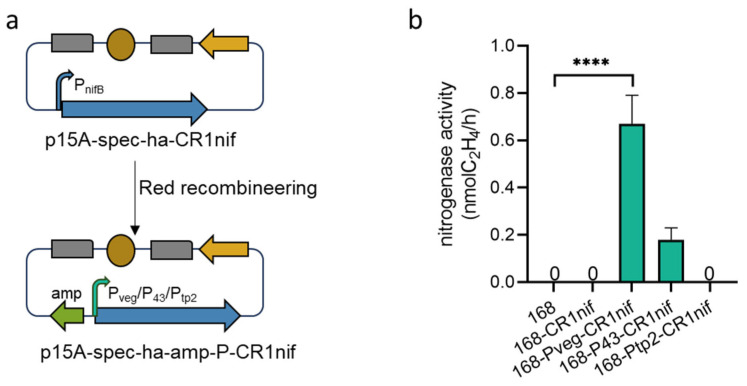
Heterologous expression of the *nif* gene cluster in *B. subtilis* 168. (**a**) Substitution of the promoter of the *nifB* gene. The native promoter of *nifB* was replaced with either the endogenous promoter P_veg_ or P_43_ or the synthetic promoter P_tp2_ via Red recombineering. (**b**) Nitrogenase activity in *B. subtilis* strains. Activity was measured as ethylene (C_2_H_4_) production (nmol/h) via the acetylene reduction assay (ARA). The tested strains include the wild-type control *(B. subtilis* 168) and engineered variants: 168-CR1nif, 168-P_veg_-CR1nif, 168-P_43_-CR1nif, and 168-P_tp2_-CR1nif. *B. subtilis* 168 (negative control) showed no detectable activity. Data represent mean values from three independent biological replicates; error bars indicate standard deviations (SDs). **** *p* < 0.0001.

Nitrogenase activity in *B. subtilis* 168-CR1nif was undetectable in the acetylene reduction assay (ARA) [22]. This implies that the ribosomal binding site (RBS) may be subjected to unidentified regulatory mechanisms. To address this, we replaced the upstream DNA sequence (including the native promoter and RBS) of the *nifB* gene with the artificial synthetic promoter P_tp2_ [23] and the endogenous promoters P_43_ and P_veg_ [24] (ordered by activity strength: P_tp2_ > P_43_ > P_veg_) (Figure 4a). Subsequently, we constructed the engineered strains *B. subtilis* 168-P_tp2_-CR1nif, 168-P_43_-CR1nif, and 168-P_veg_-CR1nif and systematically evaluated their nitrogenase activities. The results showed that the strain *B. subtilis* 168-P_veg_-CR1nif, driven by the weakest promoter, P_veg_, exhibited the highest nitrogenase activity, significantly surpassing that of *B. subtilis* 168-P_43_-CR1nif. Moreover, the strain *B. subtilis* 168-P_tp_-CR1nif, containing the strongest promoter, P_tp2_, showed no detectable activity (Figure 4b).

This phenomenon was also observed in another study, in which the refactored *nif* gene cluster from *Vibrio natriegens* was heterologously expressed in *Pseudomonas stutzeri*, and the enzymatic activity did not increase proportionally with the strength of the promoter [25]. This indicates the existence of a complex hierarchical regulatory network governing the expression of the *nif* gene cluster. Transcriptional activity can be finely modulated by trans-acting factors (e.g., σ factors and transcriptional attenuators) or post-translational modifications. Alternatively, strong promoters might drive *nifB* overexpression, causing stoichiometric imbalances between MoFe-cofactor biosynthesis and nitrogenase assembly, which in turn triggers feedback inhibition. Given the validated integrity of our cloned *nif* gene cluster and confirmed nitrogenase expression competency in *B. subtilis* 168, we propose implementing orthogonal-design-based modular refactoring of the *nif* operon in the future. This involves inserting context-optimized promoters into distinct functional modules (e.g., electron transport chains and iron–sulfur cluster assembly units) based on metabolic requirements [6,26,27]. Furthermore, integrating critical auxiliary modules (such as *nifFJ* or *nifSU*) can synergistically boost nitrogenase activity, enabling metabolic tunability in synthetic gene clusters [28].

We successfully assembled the *nif* gene cluster from *P. polymyxa* CR1 and achieved its heterologous expression in *B. subtilis* through a promoter replacement strategy. This result demonstrates that promoter selection must balance transcriptional potential with host system compatibility. This principle is particularly critical for multi-component metalloenzymes such as nitrogenase, whose functional expression necessitates the precise coordination of cofactor biosynthesis and redox homeostasis. The findings support broader engineering prospects in biofertilizer-relevant, non-model *Paenibacillus* and *Bacillus* strains. Beyond *B. subtilis*, promising host candidates include *P. polymyxa*, *B. megaterium*, and *B. licheniformis.* These strains offer distinct agricultural advantages such as rhizosphere competence, sporulation for formulation stability, or phytostimulation traits. While ethylene production confirms nitrogenase activity in vitro, future studies will employ ^15^N_2_ labeling to quantify atmospheric nitrogen assimilation and plant co-cultivation assays to evaluate agricultural relevance.

## Data Availability

The original contributions presented in this study are included in the article/Supplementary Materials. Further inquiries can be directed to the corresponding authors.

## Supplementary Materials

The following supporting information can be downloaded at https://www.mdpi.com/article/10.3390/microorganisms13061320/s1: Table S1. Strains, mutants, and plasmids in this study; Table S2. Oligonucleotide sequences used in this study. Figure S1. Centroid secondary structure of *nif* gene cluster using RNAfold 2.6.3. Figure S2. Centroid secondary structure of *nifB* gene cluster using RNAfold 2.6.3. Figure S3. Centroid secondary structure of *nifK* using RNAfold 2.6.3.

## Abbreviations

The following abbreviations are used in this manuscript:

ExoCET	Exonuclease combined with RecET recombination
ARA	Acetylene reduction assay
PGPR	Plant-growth-promoting rhizobacterium
RT-qPCR	Real-time quantitative reverse transcription
