# Supplementary material for: Heterologous Expression of the Nitrogen-Fixing Gene Cluster from Paenibacillus polymyxa in Bacillus subtilis
Source: Microorganisms. 2025 Jun 6;13(6):1320. doi: 10.3390/microorganisms13061320 (PMC12195393; doi:10.3390/microorganisms13061320)
Supplement: Supplementary file 1 [file microorganisms-13-01320-s001.zip › microorganisms-3643411-supplementary.pdf]

Supplementary Materials for

Heterologous expression of the nitrogen-fixing gene cluster from *Paenibacillus polymyxa* in *Bacillus subtilis*

Xiuling Wang†, Shiqing Gao†, Jun Fu*, and Ruijuan Li*

State Key Laboratory of Microbial Technology, Shandong University, Qingdao 266237, China

*Correspondence should be addressed to Jun Fu (fujun@sdu.edu.cn) or Ruijuan Li (liruijuan@sdu.edu.cn).

†These authors contributed equally to this work and should be considered co-first authors.

Tel. +86-532-67722918 (J.F. & R.L.); Fax +86-532-58631501 (J.F. & R.L.).

Table S1. Strains and plasmids in this study.

Strains	Genotype	Source
E. coli		
GB05-dir	GB2005, *ybcC*::P_{BAD}-ETγA	Our lab
GB05-red	GB2005, *ybcC*::P_{BAD}-αβγA	Our lab
B. subtilis		
168	Wild-type	Our lab
168-CR1nif	168, *amyE*:: CR1nif	This study
168-P_{tp2}-CR1nif	168, *amyE*:: P_{tp2}-CR1nif	This study
168-P_{43}-CR1nif	168, *amyE*:: P_{43}-CR1nif	This study
168-P_{veg}-CR1nif	168, *amyE*:: P_{veg}-CR1nif	This study
**Plasmids**		
pUC57-kan-F1	p15A replicon, *nifB*, *nifH*, kan^R	Gene synthesis
pUC57-kan-F2	p15A replicon, *nifD*, *nifK*, kan^R	Gene synthesis
pUC57-kan-F3	p15A replicon, *nifE*, *nifN*, kan^R	Gene synthesis
pUC57-kan-F4	p15A replicon, *nifX*, *hexA*, *nifV*, kan^R	Gene synthesis
pBR322-amp-tetR-tetO-ccdB-hyg	pBR322 replicon, *ccdB*, amp^R, hyg^R	Our lab
pBR322-amp-CR1nif	pBR322 replicon, *nif* gene cluster, amp^R	This study
p15A-amyEF-amp-ccdB-spec-amyER	p15A replicon, homologous arms of *amyE*, amp^R, $spec^R$	Our lab
p15A-ha-spec-CR1nif	p15A replicon, homologous arms of *amyE*, *nif* gene cluster of *P. polymyxa* CR1, $spec^R$	This study
pR6K-amp-ccdB	pR6K replicon, amp^R	Our lab
p15A-ha-spec-amp-Pveg-CR1nif	p15A replicon, *nif* gene cluster under promoter P_{veg}, homologous arms of *amyE*, amp^R, $spec^R$	This study
p15A-ha-spec-amp-P43-CR1nif	p15A replicon, *nif* gene cluster under promoter P_{43}, homologous arms of *amyE*, amp^R, $spec^R$	This study
p15A-ha-spec-amp-Ptp2-CR1nif	p15A replicon, *nif* gene cluster under promoter P_{tp2}, homologous arms of *amyE*, amp^R, $spec^R$	This study

Table S2. Oligonucleotide sequences used in this study.

Primers	Primer sequences (5'-3')
pBR322-1	cagaagggtataataaaatatagtgctgtataaagtgcgtAGATCCGAAAACCCCAAGTTACG
pBR322-2	acgttaggtaatatgtcataccatttacatgtgtgcggttaGATCCTTTCTCCTCTTTAGATC
p15A-1	gtcaaattttacgttaggtaatatgtcataccatttacatgtgtgcggttTCTTCATCATCATTGGCATACG
p15A-2	cgaagccgttcagaagggtataataaaatatagtgctgtataaagtgcgtCGAATGGCGATTTTCGTTCGTG
F1	CTTTTTATCGTCTGCGGCGG
F2	TGACTACCCTGGCTACCTCC
R1	CCGTGGAATCATCCTCCCAA
R2	CGGCGCAAATGCAGACAATA
amp-1	ctagtgacatgggtagagtcgcatgatacgtatgccaatgatgatgaagaTTACCAATGCTTAATCAGTGAGG
amp-2	TTTGTTTATTTTTCTAAATAC
veg-1	gtatttagaaaaataaacaaacAGTTGAAAACCTGCATAGGAG
veg-2	agggtttccaatgctaagggggtttccgagagatcagctaaagagtccattGCATCCACCTCACTACATTTATT
43-1	gtatttagaaaaataaacaaaTGATAGGTGGTATGTTTTCGC
43-2	ATGTGTACATTCCTCTCTTACCT
tp2-2	gttgtcaacacttttttgcgattttgggggcattataccatattttgttttgaatgtcaacacctttttgagatttaatTTGTTTATTTTTCTAAATAC
tp2-3	ttccaatgctaagggggtttccgagagatcagctaaagagtccatatgtatatctccttctttttctattataccagatctgagttaaGTTGTCAACACTTTTTTGCG
nifB-1	GCATTCATCTTCCAGTAGCC
nifB-2	AGCAGTTCACTAACAACGCC
nifK-1	GATTTGTGGCATATGCGTTCGC
nifK-2	GGATAACGATGCAGATGATGCC
nifV-1	AGCTAATGACCTGGAATCGC
nifV-2	CTGCTCAGGATTCATACCCA
gyrA-1	AACAAACATTCCTCCGCACC
gyrA-2	TTTGCCCGGATCGTGATAGA

Lower case letters are homology arms.

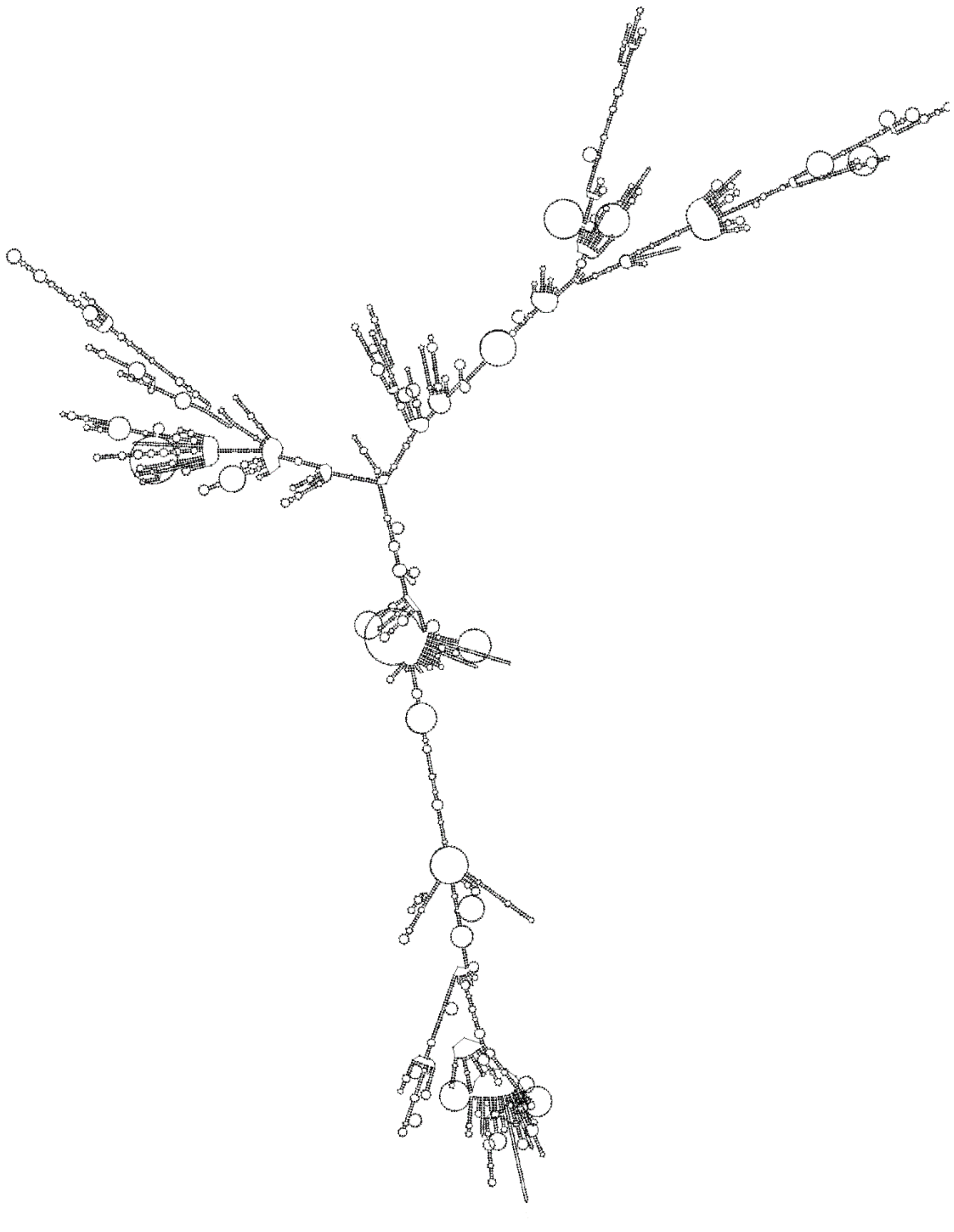

Figure S1 Centroid secondary structure of *nif* gene cluster using RNAfold 2.6.3.

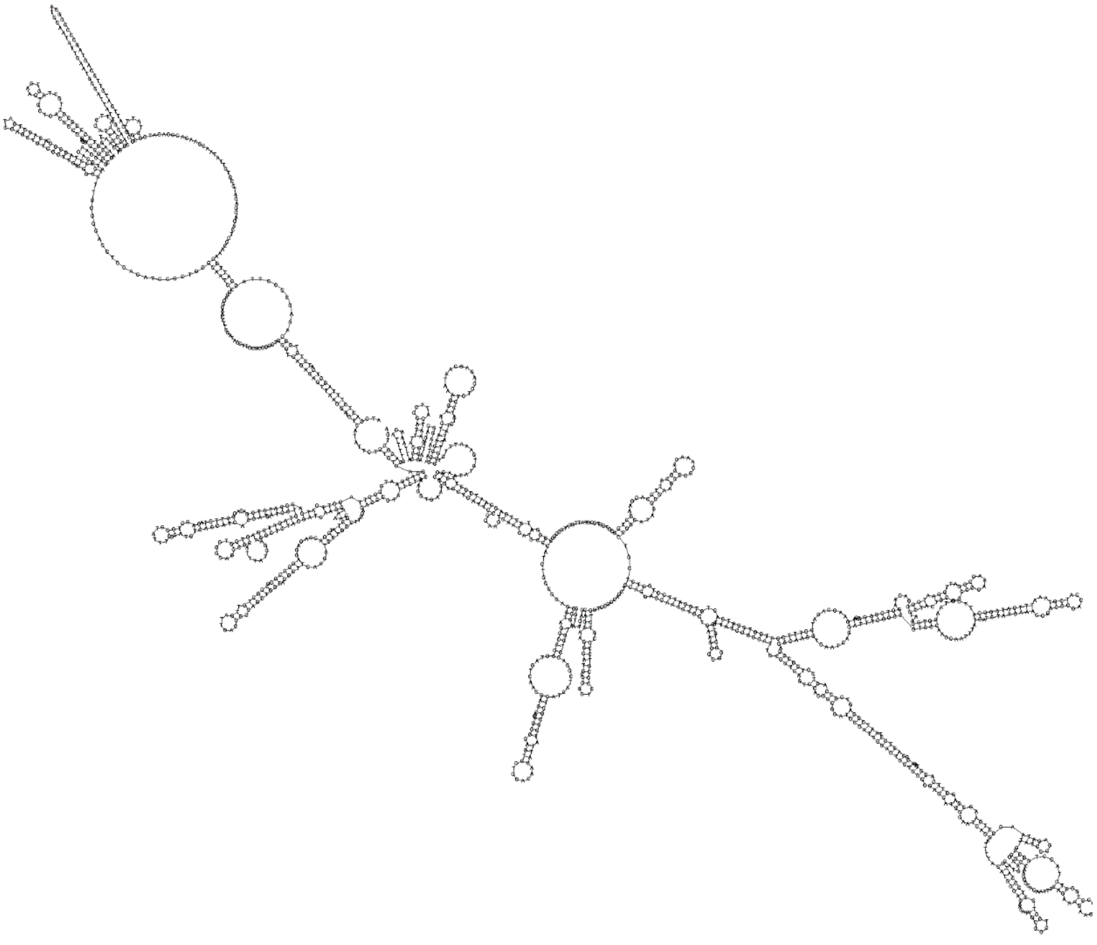

Figure S2 Centroid secondary structure of *nifB* gene cluster using RNAfold 2.6.3.

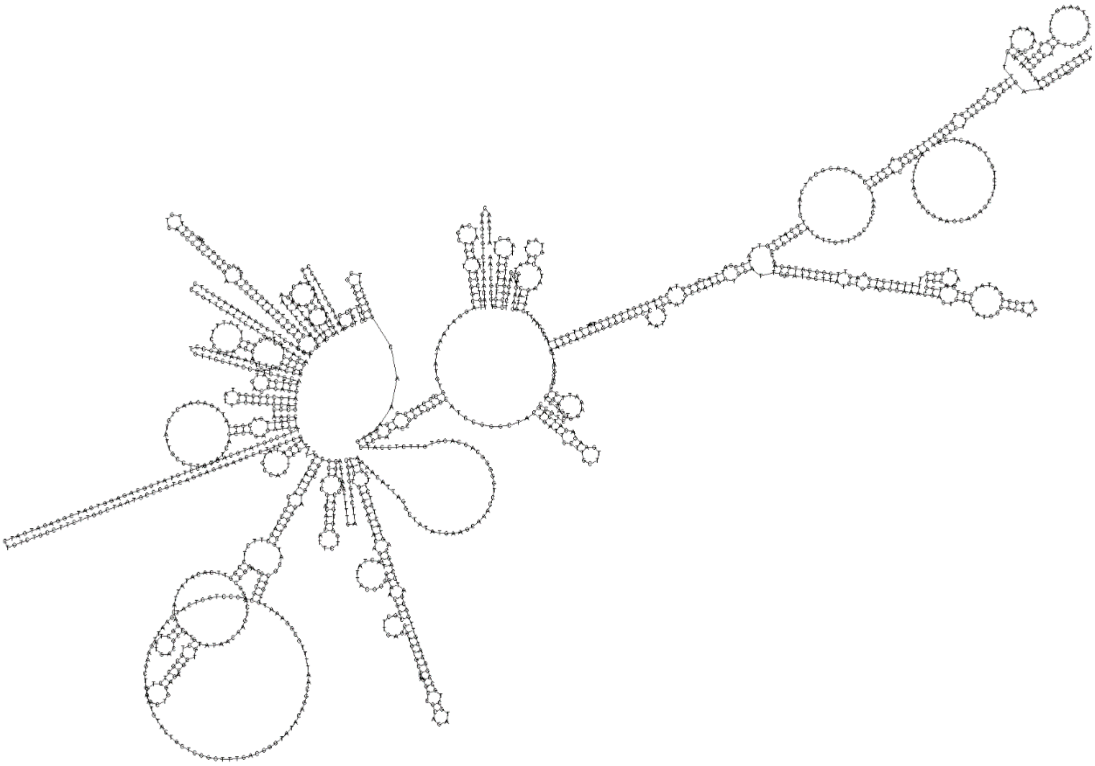

Figure S3 Centroid secondary structure of *nifK* using RNAfold 2.6.3.
